# Is Diurnal Temperature Range a Risk Factor for Childhood Diarrhea?

**DOI:** 10.1371/journal.pone.0064713

**Published:** 2013-05-28

**Authors:** Zhiwei Xu, Cunrui Huang, Lyle R. Turner, Hong Su, Zhen Qiao, Shilu Tong

**Affiliations:** 1 School of Public Health and Social Work, Institute of Health and Biomedical Innovation, Queensland University of Technology, Brisbane, Australia; 2 Centre for Environment and Population Health, School of Environment, Griffith University, Brisbane, Australia; 3 School of Public Health, Anhui Medical University, Hefei, China; The Ohio State University, United States of America

## Abstract

**Background:**

Previous studies have found that high and cold temperatures increase the risk of childhood diarrhea. However, little is known about whether the within-day variation of temperature has any effect on childhood diarrhea.

**Methods:**

A Poisson generalized linear regression model combined with a distributed lag non-linear model was used to examine the relationship between diurnal temperature range and emergency department admissions for diarrhea among children under five years in Brisbane, from 1st January 2003 to 31st December 2009.

**Results:**

There was a statistically significant relationship between diurnal temperature range and childhood diarrhea. The effect of diurnal temperature range on childhood diarrhea was the greatest at one day lag, with a 3% (95% confidence interval: 2%–5%) increase of emergency department admissions per 1°C increment of diurnal temperature range.

**Conclusion:**

Within-day variation of temperature appeared to be a risk factor for childhood diarrhea. The incidence of childhood diarrhea may increase if climate variability increases as predicted.

## Introduction

Despite an increasing number of vaccine programs implemented globally [1], diarrhea remains a major cause of morbidity and mortality worldwide among children under five years [2]. It is a big problem in developing countries [3]. While the incidence of mortality due to childhood diarrhea has been declining in some industrialized countries [4], [5], [6], it is still an important source of morbidity in these regions [7]. Considerable research of diarrheagenic pathogens has found that rotavirus is the most common cause of acute and severe childhood diarrhea, especially in industrialized countries [8], [9], [10]. In 1997 and 2000, rotavirus was reported in 18% and 19% of diarrhea-associated hospitalizations for children younger than five years in the USA, respectively [8]. Interventions in industrialized countries to improve hygiene and sanitation are more likely to affect diarrhea caused by bacteria and parasites, which are mainly transmitted through contaminated water and food. However, for rotavirus associated diarrhea which is spread from person-to person, these interventions may be less effective [11].

A complex set of environmental, nutritional, socioeconomic and cultural factors are involved in driving diarrhea prevalence [6]. In particular, daily, weekly and monthly ambient temperatures have been associated with hospital admissions for childhood diarrhea [12], [13], [14]. Yet, to date, the effect of temperature variation on childhood diarrhea has received limited attention. The temperature variation within one day, e.g., diurnal temperature range, is associated with climate change [15]. The effects of diurnal temperature range (DTR) on mortality or morbidity for cardiovascular and respiratory diseases have been studied in some Asian regions [16], [17], [18], [19]. However, few studies have examined the impact of DTR on children’s health [20], and little is known about the relationship between DTR and childhood diarrhea.

Many previous studies have reported that there is a delayed effect of DTR on human health [19], [20]. In addition, the relationship between DTR and morbidity has been postulated to be non-linear across both temperature and lag days [17]. Previous studies mainly used generalized additive model to quantify the effect of DTR on morbidity [16], [17], and manually calculated the lagged effect. A distributed lag non-linear model (DLNM) was developed to incorporate both the lagged and the non-linear effects [21]. It used a cross-basis function which describes a two-dimensional DTR-response relationship along the dimensions of DTR and lag.

This study addressed three questions: 1) What is the relationship between DTR and childhood diarrhea? 2) What is the lag structure of the effect of DTR on childhood diarrhea? 3) Between two genders, are there any differences in the magnitudes and lagged periods of DTR effect on diarrhea?

## Materials and Methods

### Data Collection

Brisbane is the capital city of Queensland, located on the east coast of Australia (27° 30′ S, 153° 00′ E). It has a sub-tropical climate, experiencing a general trend of hot summers and mild winters.

Emergency department admission (EDA) data from 1st January 2003 through 31st December 2009 were obtained from Queensland Health. Ethical approval was obtained from the Ethic Review Board of Queensland University of Technology prior to the data being collected. Because the data were de-identified and aggregated, written consent was not needed. The anonymised EDA data were classified according to the International Classification of Disease, 10th version (ICD–code 10). In this study, we included EDAs with the principle cause coded as diarrheal disease of any cause (ICD–10 codes: A00–A03, A04, A05, A06.0–A06.3, A06.9, A07.0–A07.2, A07.9, A08–A09) among children aged 0–4 years. In terms of exposure data, daily data on maximum and minimum temperatures and relative humidity in Brisbane for the same period were obtained from the Australian Bureau of Meteorology. The data were collected from a number of different stations throughout the Brisbane area, and then averaged. DTR was calculated as the daily maximum temperature minus the daily minimum temperature.

### Statistical Analyses

A Poisson generalized linear regression model combined with a distributed lag non-linear model was used to quantify the effect of DTR on childhood diarrhea. We have assessed the relationship between mean temperature and childhood diarrhea, and found there was a statistically significant relationship between them. As previous studies had suggested potential confounding effects of mean temperature, relative humidity, day-of-week, season and long-term trends on the relationship between DTR and human health [16], [19], all these factors were controlled in the model. In all cases, the Akaike Information Criterion (AIC) and analysis of residuals were used to evaluate the model fit and choice of *df*.
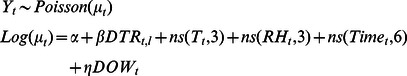



Where *t* is the day of the observation, *Y_t_* is the observed daily childhood diarrhea on day *t* and *α* is the model intercept. *DTR_t,l_* is a matrix obtained by applying the DLNM to DTR, *β* is vector of coef­ficients for *DTR_t,l_* and *l* is the lag days. The terms *ns(T_t_, 3)* and *ns(RH_t_, 3)* are natural cubic splines with three degree of freedom which control for mean temperature and relative humidity, respectively. To control for long term trends, a natural cubic spline *ns(Time_t_,6)* with six degrees of freedom was incorporated. *DOW_t_* is the categorical day of the week with a reference day of Sunday.

We found the effect of DTR on childhood diarrhea was negligible for lags above 10 days, so we calculated the relative risk of DTR with lags up to 10 days. In addition, we also used a lag-stratified approach by examining the cumulative effects of DTR at lag 0–3 days. We were interested in this period because usually diarrhea infection has an incubation period of 1–3 days [22].

All effect estimates were presented as relative risks, using a reference DTR of 10°C, which was selected by taking the average DTR level across the study period. All data analysis was conducted using the R statistical environment (version 2.15) with the “dlnm” package used to fit the regression model [23]. The compare the results, sensitivity analyses were performed by varying the *df* for time, temperature, humidity, and by altering the maximum lag in the model.

## Results

There were 11194 EDAs for diarrhea among children younger than five years across the whole study period. Table 1 shows the summary statistics for mean, maximum and minimum temperature, diurnal temperature range, relative humidity, and total, and gender-specific childhood diarrhea. The average values of mean, maximum and minimum temperature and DTR were 20.6°C (range: 9.0–34.2), 25.6°C (13.8–40.2), 15.5°C (–0.1–28.1), and 10.0°C (0.6–21.6), respectively. Every year, there were 173 days with each day’s DTR over 10.0°C, from 2003 to 2009. The average relative humidity was 57.3% (5.0%–98.0%).

**Table 1:** Summary statistics for weather variables and diarrhea in children under five years in Brisbane, Australia, from 1 January 2003 to 31 December 2009.

Variables	Mean	SD	Min	Percentile			Max
				25	50	75	
Mean temperature (°C)	20.6	4.1	9.0	17.3	20.9	23.8	34.2
Maximum temperature (°C)	25.6	3.4	13.8	23.0	25.8	28.1	40.2
Minimum temperature (°C)	15.5	5.2	−0.1	11.7	16.1	19.8	28.1
Diurnal temperature range (°C)	10.0	3.4	0.6	7.5	9.8	12.2	21.6
Relative humidity (%)	57.3	16.0	5.0	49.0	58.0	67.0	98.0
Diarrhea	4.4	3.7	0	2	4	6	27
Bacterial diarrhea	0.2	0.4	0	0	0	0	4
Viral diarrhea	2.5	2.5	0	1	2	3	22
Diarrhea in male	2.3	2.3	0	1	2	3	19
Diarrhea in female	2.1	2.0	0	1	2	3	15

The average number of daily EDAs for diarrhea was 4.4 (0–27) among children under five years in Brisbane. The daily average EDAs for virus-associated diarrhea and bacteria-associated diarrhea were 2.5 (0–22) and 0.2 (0–4), respectively.

**Figure 1:**
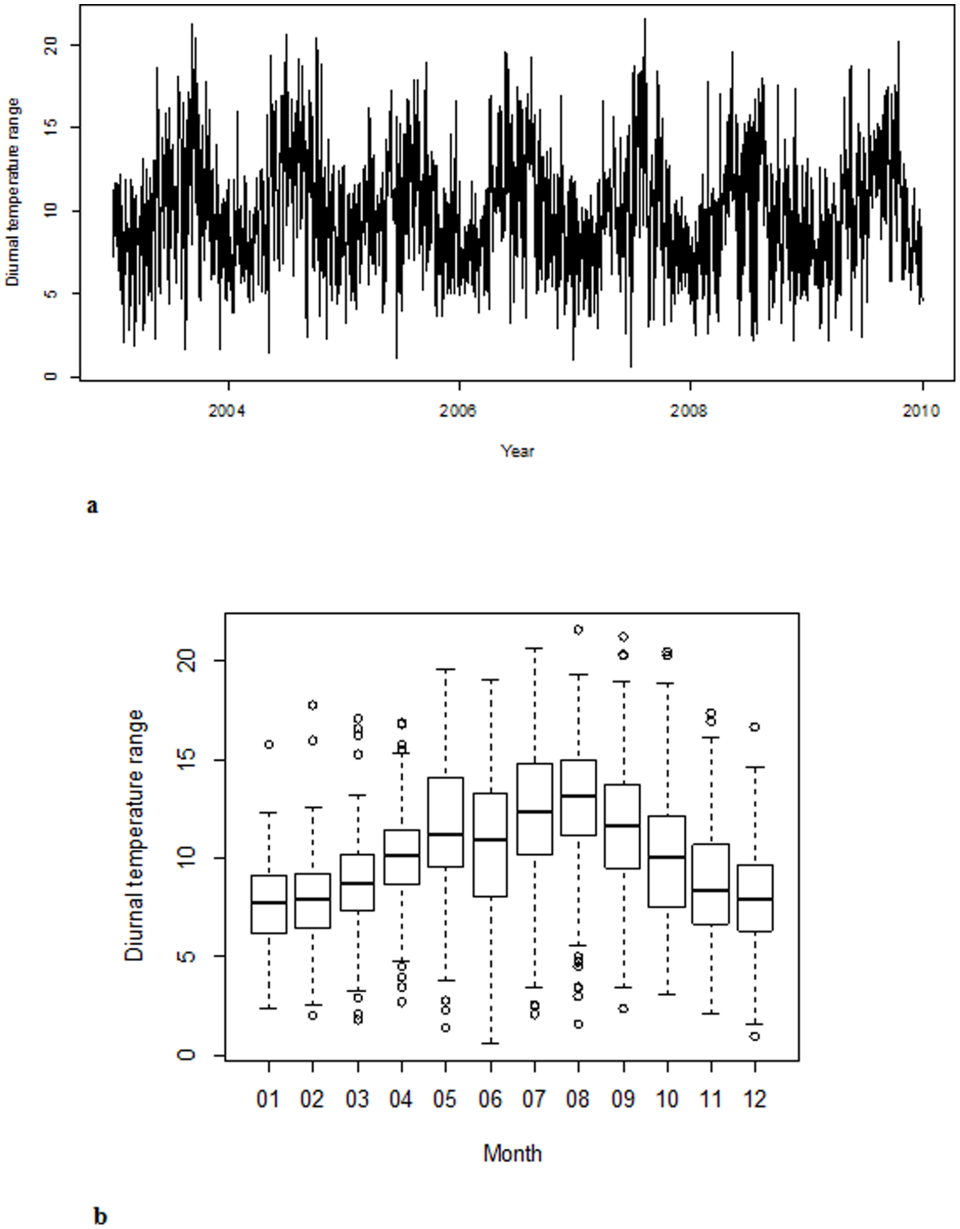
The daily and monthly distribution of diurnal temperature range.

Figure 1a presents the temporal distribution of DTR from 1 January 2003 to 31 December 2009 in Brisbane, showing a strong seasonal pattern. Figure 1b indicates that the variation of DTRs was greater in the months of May to September than other months.

**Figure 2:**
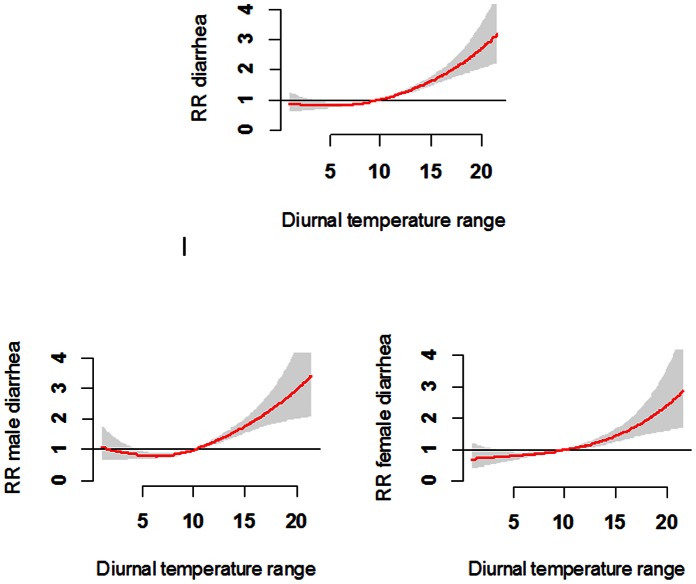
The overall effects of diurnal temperature range on diarrhea among children under five years.

Figure 2 shows the cumulative effects of DTR on the total and gender-specific childhood diarrhea. It suggests that DTR was significantly associated with childhood diarrhea. Both male and female children shared a similar DTR-diarrhea pattern, with the relative risk increasing rapidly when DTRs were over 10°C.

**Figure 3:**
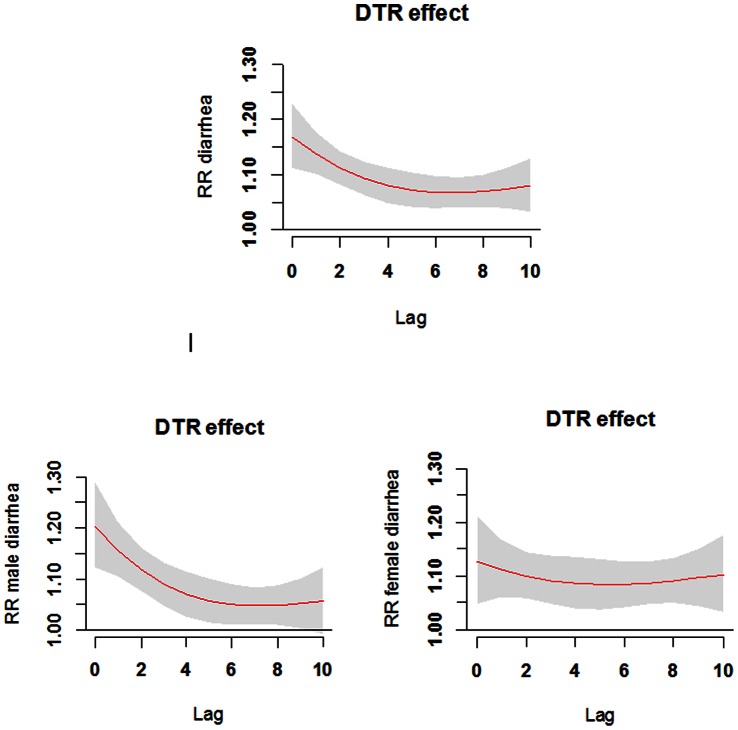
The lagged effects of diurnal temperature range on diarrhea among children under five years.

Figure 3 shows the lagged effect of DTR (95th percentile (16.1°C) relative to mean value (10.0°C)), which illustrates that the DTR effect appeared to be acute. The lagged effects of DTR on diarrhea differed between males and females. The DTR effect on the male children was greater on the first day after exposure when compared with females, but it decreased rapidly within ten days, while the DTR effect on female children lasted longer.

Table 2 depicts the association between DTR and diarrhea in children under five years, suggesting that the effect was the greatest at one day lag. In particular, a 1°C increase in diurnal temperature range was associated with a 3% (95% CI: 2%, 5%) increase of EDAs for childhood diarrhea.

**Table 2:** Relative risk (RR) of diarrhea in children under five years associated with one unit increase in diurnal temperature range.

	RR (95% CI)				
	Lag 0	Lag 1	Lag 2	Lag 3	Lag 4–10
Total	1.01(0.99,1.02)	1.03(1.02,1.05)*	1.03(1.01,1.04)*	1.02(1.01,1.04)*	1.00(0.99,1.01)
Male	1.02(0.99,1.04)	1.02(0.99,1.04)	1.03(1.01,1.05)*	1.03(1.01,1.05)*	1.00(0.99,1.01)
Female	1.00(0.97,1.02)	1.03(1.01,1.05)*	1.03(1.01,1.05)*	1.02(0.99,1.04)	1.01(0.99,1.02)

* P-value<0.05; R^2^ = 0.6994; Adjusted R^2^ = 0.6792.

**Figure 4:**
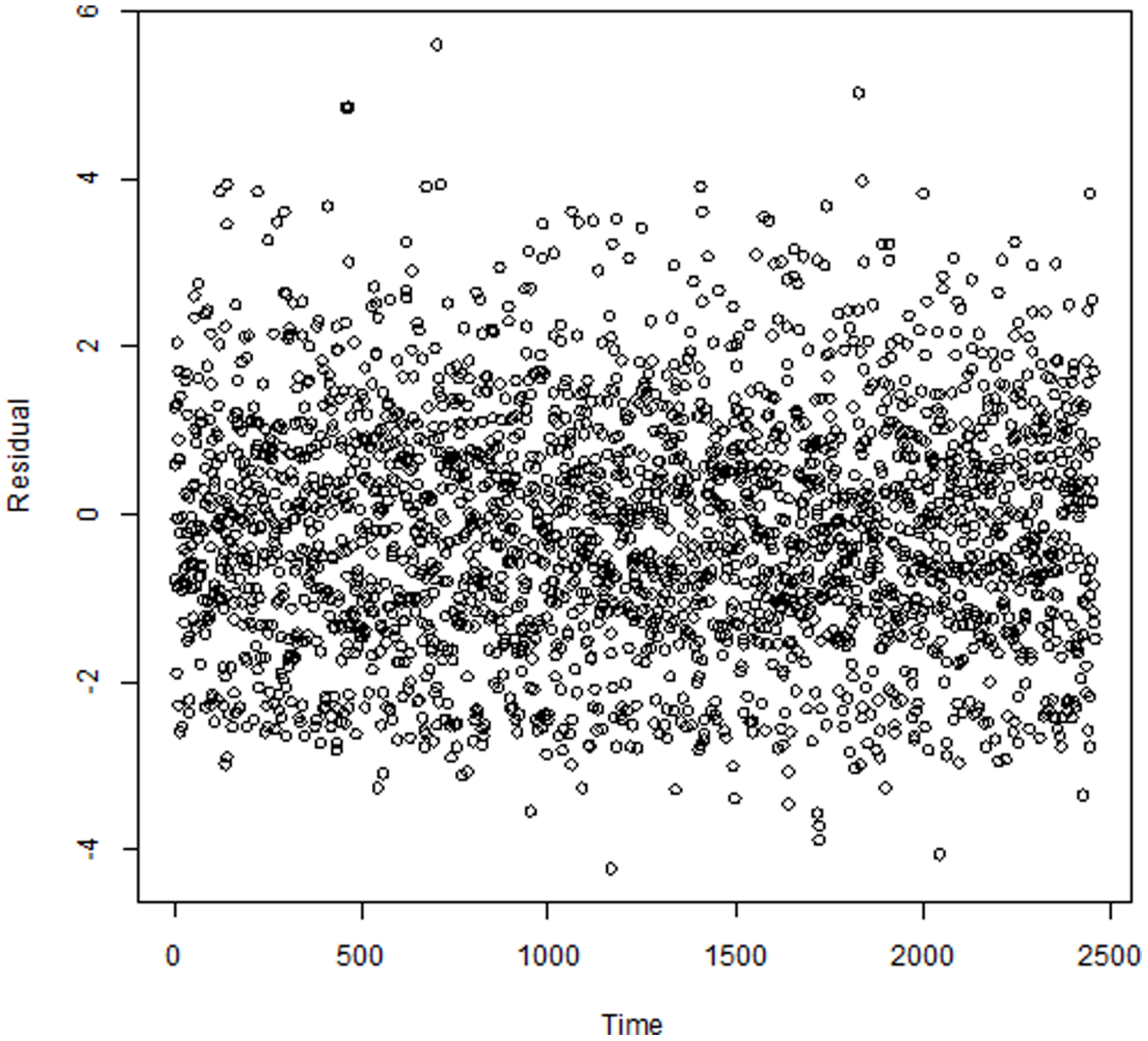
The scatter plot of residuals over time.

The residuals were checked to evaluate the adequacy of the model (Figure 4). Sensitivity analysis was conducted by changing the *df* (7–15 per year) for time to control for season. We also varied the *df* (4–7) for temperature, humidity, and altered the maximum lags from 11 to 20 days for the DLNM. We found that the results changed little (results not shown).

## Discussion

Our study examined both the non-linear nature and lagged effects of DTR on childhood diarrhea, and found that there was a significant relationship between DTR and childhood diarrhea. Every year between 2003 and 2009, children in Brisbane were exposed to the risk of relatively large DTR (>10°C) for over 170 days. The DTR effect on childhood diarrhea was acute, with the greatest effect at one day lag.

The results of this study support our hypothesis that there was a significant association between DTR and childhood diarrhea. Currently, the exact mechanism by which exposure to a large DTR can increase the risk of childhood morbidity remains largely unknown. Bull argued that sudden changes in weather conditions may affect either humoral or cellular immunity [24]. Very young children have a relatively immature immune system [25] and low self-care capacity [26], which might result in a greater vulnerability to temperature change. Studies have found that sudden changes in the temperature of inhaled air are associated with the release of inflammatory mediators by mast cells [27], [28], which could also be related to higher diarrhea prevalence [29], [30]. Further study is necessary to determine which biomarkers are affected by DTR.

In this study, the DTR effect increased rapidly above 10°C (average level of DTR across the whole study period), highlighting that both parents and medical staff should be made aware of the particularly high risk posed by large DTR and diarrhea-related morbidity in children. In Brisbane during the study period, children were exposed to the risk of relatively large DTR for more than 170 days every year. In terms of the lag structure of the effect of DTR, we found that the greatest effect was at one day lag, which is inconsistent with previous studies examining the relationship between DTR and mortality caused by chronic obstructive pulmonary disease [19]. This suggests that caution is necessary when generalizing the results of this study directly to other disease types or target populations.

Interestingly, in this study, we found that male and female children shared the similar general DTR-diarrhea pattern. So far, only two studies have examined the gender differences in vulnerability to DTR effect [17], [31], and they found that the modification effect of gender on DTR was not distinct, which is consistent with our results. However, the lagged effect pattern of DTR on male children was quite different from that of female children, indicating that caregivers of female children should be made aware of the longer risk of big DTRs. The reasons for why DTR effects on female children lasted longer is currently unclear. It might be due to the variations in body composition (e.g., sexual dimorphism) [32], [33] and social behaviour (e.g., daily activity). Basu argued that differences in the effect of temperature on males and females varied among different locations and populations [34].

This study has several strengths. This is, to our knowledge, the first study to examine the association between DTR and childhood diarrhea, while the data on the wider relationship between DTR and pediatric morbidity are limited in the literature. Advanced statistical methods were used to quantify the non-linear and lagged effects of DTR on childhood diarrhea. We elucidated the lag structure of DTR on childhood diarrhea, considering lagged effects up to 10 days.

This study also has several limitations. Firstly, we only included data from one city, which means that our findings should be generalised to other regions with caution. However, the findings from this study may encourage future research in other populations. Secondly, there might be some exposure misclassification bias because we used aggregated data on temperature rather than individual exposure data. Thirdly, some factors at individual level may have impact on the relationship between diurnal temperature range and diarrhea, but we do not have identifiable individual information, so we cannot analyse the effect of person to person contact on diarrhea in this study. Fourthly, there is a possibility that a child with diarrhea might go to hospital twice in ten days. Though we think that the number of such cases is very small, especially in such a short period of time, there still might be some biases caused by this.

### Conclusions

Our study reveals a significant association between DTR and diarrhea among children younger than five years. The effect of DTR on childhood diarrhea was greatest at a lag of one day. It is important for both parents and health practitioners to be aware of the impact of DTR on diarrhea in children, and take protective measures to reduce the associated risks when DTRs increase. As climate change continues, DTRs are likely to become more variable. Therefore, the associated health impacts are also likely to increase, particularly among the vulnerable populations including children. More research and targeted health policies and programs are needed to identify and minimize these risks.
